# Many Facets of Eating Disorders: Profiling Key Psychological Features of Anorexia Nervosa and Binge Eating Disorder

**DOI:** 10.3390/bs13030276

**Published:** 2023-03-21

**Authors:** Alessandro Alberto Rossi, Giada Pietrabissa, Andrea Tagliagambe, Anna Scuderi, Lorenzo Montecchiani, Gianluca Castelnuovo, Stefania Mannarini, Laura Dalla Ragione

**Affiliations:** 1 Department of Philosophy, Sociology, Education, and Applied Psychology, Section of Applied Psychology, University of Padua, 35131 Padua, Italy; 2 Interdepartmental Center for Family Research, University of Padua, 35131 Padua, Italy; 3 Department of Psychology, Catholic University of Milan, 20123 Milan, Italy; 4 Clinical Psychology Research Laboratory, Ospedale San Giuseppe, IRCCS Istituto Auxologico Italiano, 28824 Verbania, Italy; 5 Residence Cabrini DCA, 54027 Pontremoli, Italy; 6 Eating Disorders Services-USL N1 “Nido delle Rondini”, 06059 Todi, Italy; 7 Eating Disorders Services-USL N1 “Palazzo Francisci”, 06059 Todi, Italy; 8 Food Science and Human Nutrition Unit, University Campus Biomedico of Rome, 00128 Rome, Italy

**Keywords:** eating disorders, eating behaviors, binge eating disorder, anorexia nervosa, profile analysis

## Abstract

*Objective*. The present study employs a profile analysis to identify and compare psychological features and core eating disorder (ED) symptoms in clinical samples of patients with anorexia nervosa (AN) and binge eating disorder (BED) and the general population (GP). *Methods.* A sample comprising 421 participants (142 patients with AN; 139 patients with BED; and 140 participants from the GP) was surveyed with the Eating Disorder Inventory-3 (EDI-3). Individuals with AN and BED were recruited and tested during their first week of a multidisciplinary inpatient program for weight loss and rehabilitation at the ‘Rete DCA USL Umbria 1′ (Eating Disorders Services), Italy. *Results*. The findings suggest distinct patterns of symptom presentation between the three samples across all the EDI-3 dimensions—with both the AN and BED groups scoring significantly higher than the GP. Patients with AN registered greater scores in all the psychological trait scales and the drive for thinness ED-specific dimension of the EDI-3 compared with their BED counterpart—which, instead, scored higher in the bulimia and body dissatisfaction subscales. These data support the transdiagnostic nature of the main risk factors for the onset and maintenance of EDs—which would vary in severity levels—and the existence of disease-specific pathways giving rise to AN and BED. *Conclusion*. This study for the first time compares patients with AN and BED with a non-clinical sample on main ED psychological features. This might inform classification approaches and could have important implications for the development of prevention and early intervention programs.

## 1. Introduction

Feeding and eating disorders (EDs) are mental health conditions characterized by abnormal patterns of eating behavior and negative thoughts and feelings about food and one’s body [1].

International epidemiological research in Western countries indicated a lifetime prevalence of EDs as follows: anorexia nervosa (AN) at 0.16%; bulimia nervosa (BN) at 0.63%; and binge eating disorder (BED) at 1.53% [2,3].

AN is characterized by an intense fear of gaining weight or becoming fat and distorted perceptions of weight or body shape, which motivates severe dietary restriction or other weight-loss conducts despite starvation (i.e., purging, fasting, or over-exercising) leading to abnormally low body weight [1]. BN is marked by distorted body image and an obsessive desire to lose weight, in which uncontrolled episodes of overeating are followed by self-induced vomiting, misuse of laxatives, and other methods designed to compensate for the effects of binge eating. Similarly, people with BED consume large amounts of food and feel unable to stop eating but in absence of compensatory behaviors. This commonly leads the persons to experience negative emotions—including guilt, shame, or depression—and to gain weight as a result of restrained eating because of over-compensatory overeating during lapses [4,5].

Despite the diagnostic need to define the various manifestations of dysfunctional eating behaviors as discrete categories that are qualitatively different from one another, taxonometric research—designed to classify the latent structure of phenomena—showed that changes in eating behavior designed to control body weight are often observed to varying degrees in all individuals. It also documented that EDs hallmarks can manifest themselves across different diagnoses rather than only in distinct categories [6,7,8].

Still, the existence of a dimensional latent structure of EDs had already been proposed in earlier research using discriminant function analysis [9], and, more recently, a meta-analysis conducted by Markon et al. (2011) found adequate empirical and theoretical evidence sustaining that a dimensional view of psychopathology is more reliable and valid than a categorical one [10].

Indeed, longitudinal studies showed that individuals with an ED might move between diagnostic states over time.

For example, Tozzi et al. (2005) showed that 32 out of 88 individuals diagnosed with a restricting-type AN developed BN—91% of which within the first 5 years. Additionally, in a sample of 350 subjects with BN, 27% developed AN, binge-eating/purging type—77% of which within the first 5 years [11]. Similarly, a subsequent investigation found that most women diagnosed with AN experienced diagnostic crossover to BN: during 7 years, over half of them oscillated between the restricting and binge eating/purging AN subtypes; one-third crossed over to BN [12].

Furthermore, in a study examining the course of the full range of EDs across 3 time points over 30 months was observed that only one-third of a total sample of 192 women (55 with AN, 108 with BN, and 29 suffering from an eating disorder not otherwise specified) retained their original diagnosis [13]. Diagnostic instability also characterized a large clinical sample of patients with various EDs (n = 793) whose symptomatologic course was evaluated in a 6-year follow-up study. The presence of mood disorders was found to be a significant cause of crossover between the diagnoses, and shape concern severity was a significant modifier outcome [14].

Taken together, these findings suggest that EDs may not be distinct but are rather overlapping and interrelated, and that common processes might be involved in their etiopathogenesis and persistence over time.

This is in line with the transdiagnostic conceptualization of EDs [15], according to which four core maintaining mechanisms would operate across all EDs diagnostic categories. These include (1) extreme perfectionism, defined as the over-evaluation of the striving for and achievement of personally demanding standards despite adverse consequences; (2) the difficulty of coping with intense mood states; (3) the impact of low self-esteem that motivates individuals to pursue achievement in the valued domain of weight and shape control to increase feelings of self-worth; and (4) marked interpersonal difficulties, which may lead to increased dietary restraint to control weight and shape and achieve the perceived socially valued ideal. The transdiagnostic theory has received empirical support [16,17,18]. Additionally, Olatunji, Kim, and Wall (2015) [19] proposed an important extension of this model by recognizing body thinness, body perfectionism, and body awareness as common symptom dimensions across diagnostic categories.

Focusing on common symptom dimensions across eating disorder diagnoses, rather than specific diagnoses, could advance current knowledge on mechanisms of etiology and maintenance of EDs. It would also help to improve their treatment and to inform the selection of more reliable and valid symptom measures [20,21].

Given these considerations, the present study made use of a profile analysis to compare symptoms and psychological features of EDs between two clinical samples of inpatients with AN and BED and the general population.

Indeed, while several studies assessed and compared samples of patients with AN and BN [12,22,23,24,25] or BN and BED presentations [26,27,28,29,30,31,32,33], no research to our knowledge has yet compared psychological features of individuals diagnosed with AN with those presenting BED psychopathology [34]. Further, studies have also been usually conducted on clinical samples and do not, therefore, provide rigorous empirical comparisons of psychological characteristics between individuals with an ED and a community sample.

To extend research on EDs, the current study for the first time provides a direct assessment and comparison of the Eating Disorder Inventory-3 (EDI-3) dimensions between inpatients with AN and BED and respondents from the general population to identify distinct patterns of symptom profiles that may inform the development of early intervention programs.

## 2. Methods

A cross-sectional design has been employed for this study.

### 2.1. Procedures

A survey containing a socio-demographic report form and the EDI-3 was administered to each participant.

Individuals with AN and BED were recruited at the ‘Rete DCA USL Umbria 1′ (Eating Disorders Services), Italy, during the first week of a multidisciplinary inpatient program for weight and psychological rehabilitation. The survey was administered individually in a dedicated room by a clinical psychologist working in the clinic and independent of the study as part of the routine clinical assessment.

In line with previous studies [35,36], participants from the GP were enrolled using the snowball sampling technique, personal invitations, and advertisements placed in the Universities of Padua and Milan, cafés, and libraries.

Each participant voluntarily agreed to participate in the study and signed the written informed consent.

The research project was previously approved by the Ethics Committee of the Azienda Ospedaliera di Perugia (CER Umbria): protocol n°: 22877/21/ON.

### 2.2. Sample Size Determination

The minimum sample size required for this study was computed a priori by using the G*Power software [37]. Given the main analysis of the study (see dedicated section), the multivariate analysis family of statistics was chosen—specifying 3 independent groups of patients (AN vs. BED vs. GP) and 12 different psychological scales as response variables (see ‘instruments’ section) [38,39,40,41,42]. According to guidelines [43], a priori statistics were set to small values (small effects): Pillai’s trace (V) was set to 0.2 (effects provide a minimum contribution [44])—resulting in a small effect size: *f*^2^(V) = 0.11 [43,45]. Additionally, according to general guidelines [43], the Type I error (α) rate was set at 0.05 (two-sided), and the Power (1—β) was set at 0.95. Results showed that there is more than a 95% chance of correctly rejecting the null hypothesis of no significant effect of the interaction with an overall sample of 159 subjects—53 participants per group.

### 2.3. Participants

Patients referring to the ‘Rete DCA USL Umbria 1′ (Eating Disorders Services), Italy, were consecutively recruited and screened for admission into the study within the first week of a multidisciplinary inpatient program for weight and psychological rehabilitation.

Inclusion criteria were (1) being a native Italian speaker; (2) being 18 years or older; (3) being diagnosed with an ED (AN or BED) within the last 6 months according to the DSM-5 criteria [1,45] by clinical professionals; and (4) providing signed informed consent.

Unlike the clinical sample, participants enrolled from the general population (GP) were required to have never been diagnosed with an ED (AN, BED, BN, etc.).

Participants were excluded from the study if (1) unable to complete the assessment procedure due to cognitive or vision impairments and/or illiteracy; (2) provided missing data responses.

### 2.4. Measures

*Demographics and clinical* information included age, gender, weight (in kg), height (in meters)—used to calculate the individuals’ body mass index (BMI), familiarity with EDs, previous psychological treatments, and previous hospitalizations for EDs,

Moreover, the Italian version of the *Eating Disorder Inventory-3 (EDI-3)* was administered to the participants in the study. The EDI-3 is one of the most used self-report questionnaires for a clinical evaluation of the presence and intensity of psychological traits and symptomatology associated with EDs [20,21]. The EDI-3 is composed of 91 items rated on a six-point Likert scale ranging from A (=“always”) to F (=“never”). Higher scores reflect higher levels of the specific measured construct. The EDI-3 is organized into 12 primary scales, consisting of 3 eating disorder-specific scales (Drive for Thinness—DT; Bulimia—B; and Body Dissatisfaction—BD) and 9 general psychological scales that are highly relevant to but not specific to EDs (Low Self-Esteem—LSE; Personal Alienation—PA; Interpersonal Insecurity—II; Interpersonal Alienation—IA; Interoceptive Deficits—ID; Emotional Dysregulation—ED; Perfectionism—P; Asceticism—A; Maturity Fears—MF). These 12 scales yield 6 composite scores: 1 specific Eating Concerns Composite (ECC) and 5 general integrative psychological constructs (Ineffectiveness Composite—IC; Interpersonal Problems Composite—IPC; Affective Problems Composite—APC; Overcontrol Composite—OC; and Global Psychological Maladjustment—GPM). In this study, the 12 primary scales were considered.

#### 2.4.1. Drive for Thinness (DT)

Drive for thinness (DT) is one of the main features of ED—especially for AN and BN but also for BED—and it has been considered an essential criterion for a diagnosis of EDs. The DT scale assesses an extreme desire to be thinner, concern with dieting, preoccupation with weight, and an intense fear of weight gain. The DT scale is composed of seven items. In this study, the DT scale showed good internal consistency: Cronbach’s alpha = 0.915; McDonald’s omega = 0.917.

#### 2.4.2. Bulimia (B)

The Bulimia (B) scale evaluates the predisposition to think about and engage in binge eating episodes. The B scale assesses worries about overeating and eating in response to being emotionally upset. The tendency to engage in uncontrollable overeating is common in individuals with a diagnosis of BED and BN, but it is also recurrent (but less severe) in individuals who do not meet the criteria for a diagnosis of an ED. The B scale is composed of eight items. In this study, the B scale showed good internal consistency: Cronbach’s alpha = 0.873; McDonald’s omega = 0.878.

#### 2.4.3. Body Dissatisfaction (BD)

The Body Dissatisfaction (BD) scale measures discontent with both the shape and the size of those regions of the body such as thighs, hips, stomach, etc. Even in this case, body dissatisfaction is common in individuals with a diagnosis of ED but it is also frequent (but less severe) in individuals who do not meet the criteria for a diagnosis of an ED. The B scale is composed of 10 items. In this study, the BD scale showed good internal consistency: Cronbach’s alpha = 0.861; McDonald’s omega = 0.864.

#### 2.4.4. Low Self-Esteem (LSE)

The Low Self-Esteem (LSE) scale assesses self-devaluation (namely, negative self-evaluation), which has a major role in the development and maintenance of EDs. The LSE scale evaluates feelings of insecurity, inadequacy, ineffectiveness, and lack of personal worth. The LSE scale is composed of six items. In this study, the LSE scale showed good internal consistency: Cronbach’s alpha = 0.907; McDonald’s omega = 0.910.

#### 2.4.5. Personal Alienation (PA)

The Personal Alienation (PA) scale assesses a pervasive sense of poor self-understanding, emotional emptiness, and solitude. Moreover, the PA scale evaluates the desire to be someone else and the feeling of being out of control of things in one’s own life. The PA scale is composed of seven items. In this study, the PA scale showed good internal consistency: Cronbach’s alpha = 0.844; McDonald’s omega = 0.846.

#### 2.4.6. Interpersonal Insecurity (II)

The Interpersonal Insecurity (II) scale evaluates embarrassment, uneasiness, and reticence in social situations—focusing on difficulties expressing personal thoughts and feelings in a social context as well as the tendency to withdraw and isolate from others. The II scale is composed of seven items. In this study, the II scale showed good internal consistency: Cronbach’s alpha = 0.834; McDonald’s omega = 0.838.

#### 2.4.7. Interpersonal Alienation (IA)

The Interpersonal Alienation (IA) scale measures detachment, discontent, estrangement, and lack of trust in others. The IA scale evaluates the propensity to feel imprisoned in relationships as well as the sense that there is a lack of understanding and love from others. The IA scale is composed of seven items. In this study, the IA scale showed good internal consistency: Cronbach’s alpha = 0.769; McDonald’s omega = 0.772.

#### 2.4.8. Interoceptive Deficits (ID)

The Interoceptive Deficits (ID) scale assesses two important characteristics of those who develop EDs: (a) the distress triggered by too strong and/or uncontrollable emotions (namely, ‘fear of affect’) and (b) the difficulty in correctly recognizing emotional states (namely, ‘affective confusion’). The ID scale is composed of nine items. In this study, the ID scale showed good internal consistency: Cronbach’s alpha = 0.898; McDonald’s omega = 0.899.

#### 2.4.9. Emotional Dysregulation (EmoD)

The Emotional Dysregulation (EmoD) scale measures the propensity to impulsivity, mood instability, anger, self-destructiveness, and recklessness. The predisposition toward reduced impulse regulation and mood intolerance has been identified as poor prognostic signs in EDs. The EmoD scale is composed of eight items. In this study, the EmoD scale showed good internal consistency: Cronbach’s alpha = 0.807; McDonald’s omega = 0.843.

#### 2.4.10. Perfectionism (P)

The Perfectionism scale (P) assesses the degree to which an individual gives importance to reaching high standards of personal accomplishment through severe personal standards for performance and/or pressure from parents and teachers. Perfectionism is considered a fundamental feature for the development and maintenance of EDs—mainly for AN. The P scale is composed of six items. In this study, the P scale showed good internal consistency: Cronbach’s alpha = 0.759; McDonald’s omega = 0.733.

#### 2.4.11. Asceticism (A)

The Asceticism (A) scale measures the propensity to pursue virtue through the striving for spiritual ideals such as self-discipline, self-denial, self-control, self-sacrifice, and control of bodily impulses. The A scale is composed of seven items. In this study, the A scale showed good internal consistency: Cronbach’s alpha = 0.766; McDonald’s omega = 0.776.

#### 2.4.12. Maturity Fears (MF)

The Maturity Fears (MF) evaluates the desire to return to the safety of childhood, avoiding the confusion, conflict, and developmental expectations associated with adulthood—which can stimulate fears related to role changes for which the person feels unprepared. The MF scale is composed of eight items. In this study MF scale showed good internal consistency: Cronbach’s alpha = 0.833; McDonald’s omega = 0.839.

### 2.5. Statistical Analyses

Data analysis was performed by using R software and the following packages: ‘esvis’ [46], ‘ggplot2′ [47], ‘profileR’ [38,48], ‘psych’ [49], and tidyverse [50].

First, according to the guidelines [40], the normality, linearity, multicollinearity, and homogeneity of covariance matrices were inspected.

Second, a profile analysis was performed. Profile analysis (a special case of MANOVA) allows to both determine, quantify, and interpret the extent to which the three groups of individuals (independent variable) revealed different profiles on variables implied in EDs (dependent variables)—quantifying the degree of dissimilarity between profiles [38,48,51,52,53]. According to the guidelines, before performing profile analysis, all dependent variables were rescaled into *z*-scores [38,40]. Profile analysis is a multivariate approach to test mean differences toward three specific statistics: (I) *parallelism*; (II) *level equality*, and (III) *flatness* [39,40,53,54]. (I) *Parallelism* assesses whether the shape of two profiles is analogous and symmetrical (parallel) between different groups—between-subject general statistics. To assess for parallelism, 11 segments were artificially created: (1) DT vs. B; (2) B vs. BD; (3) BD vs. LSE; (4) LSE vs. PA; (5) PA vs. II; (6) II vs. IA; (7) IA vs. ID; (8) ID vs. EmoD; (9) EmoD vs. P; (10) P vs. A; and (11) A vs. MF. Each segment represents the slope of the line among the means of two close variables, and slopes are used to test whether the difference between two segments is the same across groups. Wilks’ lambda (Λ) was chosen to test the multivariate effect. (II) *Level equality* refers to the degree of similarity in means of scores across all of the dependent variables across all groups—general between-subject statistic. To test level equality, several focused comparisons between groups were performed [55]. Finally, (III) *Flatness* aimed to determine whether (within each profile) each variable score yielded a similar response to the following variable—general within-subjects statistic [40]. To test flatness, several focused repeated measures comparisons were also performed for each group to assess within-group effects.

Bonferroni’s correction was applied. Partial eta-square (η^2^_p_) and Cohen’s *d* were used to quantify the difference in multiple and pairwise comparisons, respectively—with the following benchmarks: small (η^2^_p_: 0.011 to 0.059; *d*: 0.20 to 0.49), moderate (η^2^_p_: 0.060 to 0.139; *d*: 0.50 to 0.79), and large (η^2^_p_ > 0.140; *d* > 0.80) [40,42].

## 3. Results

### 3.1. Sample Characteristics

The final sample comprised 421 participants. Patients with AN were 142 [5 males (3.5%) and 137 females (96.5%)], were aged from 18 to 65 years (*mean* = 27.96, *SD* = 10.64), and had a BMI ranging from 11.40 to 18.42 Kg/m^2^ (*mean* = 15.21, *SD* = 1.63). Additionally, 80% of them had no familiarity with a diagnosis of ED and reported no previous treatments or hospitalizations for an ED. Patients with BED were 139 [33 males (23.7%) and 106 females (76.3%)], were aged from 18 to 70 years (*mean* = 44.65, *SD* = 15.21), and had a BMI ranging from 23.00 to 71.47 Kg/m^2^ (*mean* = 41.54, *SD* = 9.53). Furthermore, 77.4% of them had no familiarity with a diagnosis of ED, and 80.4% of the respondents reported no previous treatments or hospitalizations for an ED. Participants from the GP were 140 [33 males (25%) and 105 females (75%)], were aged from 18 to 70 years (*mean* = 42.30, *SD* = 14.35), and had a BMI ranging from 15.24 to 65.79 Kg/m^2^ (*mean* = 23.70, *SD* = 5.51). None of them had familiarity with a diagnosis of ED, previous treatments, or hospitalizations for an ED.

### 3.2. Preliminary Analyses

First, univariate normality was assessed. As reported in Table 1, the raw score of each dependent variable was almost normally distributed. Second, the linearity of bivariate relationships among dependent variables was observed using a scatter matrix that revealed no curvilinear relationships. Multicollinearity among dependent variables was assessed using Pearson’s bivariate correlation coefficients, tolerance, and variance inflation factor (VIF) statistics—which revealed the absence of multicollinearity (Table 1). Finally, the homogeneity of variance–covariance matrices was tested through the Box’s *M* test—which resulted to be statistically significant (*M* = 466.788, *F* = 2.873, *p* < 0.001); however, it should be noted that this statistic is overpowered when groups have equal size—as in this case [40]. Thus, considering these results, a profile analysis was performed [40,55].

### 3.3. Profile Analysis: Parallelism

**Figure 1:**
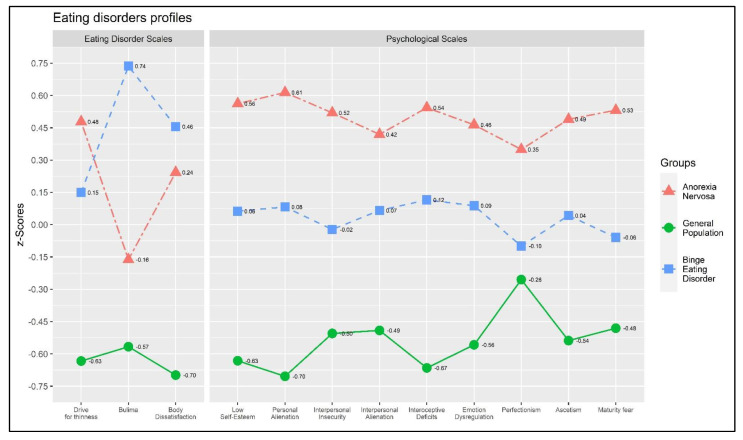
The plot of the profile analysis. *Note*: Eating disorder scales: Drive for Thinness—DT; Bulimia—B; and Body Dissatisfaction—BD. General psychological scales: Low Self-Esteem—LSE; Personal Alienation—PA; Interpersonal Insecurity—II; Interpersonal Alienation—IA; Interoceptive Deficits—ID; Emotional Dysregulation—ED; Perfectionism—P; Asceticism—A; Maturity Fears—MF. Participants with anorexia nervosa: *n* = 142; participants with binge eating disorder: *n* = 139; participants from the general population: *n* = 140.

A statistically significant interaction effect between the three groups (AN vs. BED vs. GP) and the selected EDs-related variables was found—showing an absence of parallelism among profiles: Wilks’ Λ = 0.549, *F* = 12.983, *p* < 0.001, η^2^_p_ = 0.259 (large effect size). This revealed that segments were different across conditions. Figure 1 graphically represents the absence of parallelism.

### 3.4. Profile Analysis: Level Equality—Between-Group Differences

A statistically significant between-groups effect was found: *F* = 93.47, *p* < 0.001; η^2^_p_ = 0.309 (large effect size). This outcome further confirmed that the three groups (AN vs. BED vs. GP) were different, on average.

Multivariate pairwise focused contrast between the AN group and the GP showed a statistically significant multivariate effect [Wilks’s Λ = 0.798, *F* = 6.248, *p* < 0.001, η^2^_p_ = 0.202 (large effect size)] and a statistically significant between-groups difference [*F* = 179.446, *p* < 0.001, η^2^_p_ = 0.391 (large effect size)]. Moreover, multivariate pairwise focused contrast between the GP and the BED group presented a statistically significant multivariate effect [Wilks’s Λ = 0.670, *F* = 11.968, *p* < 0.001, η^2^_p_ = 0.330 (large effect size)] and a statistically significant between-groups difference [*F* = 105.410, *p* < 0.001, η^2^_p_ = 0.276 (large effect size)]. Lastly, a multivariate pairwise focused contrast between the AN and BED conditions exhibited a statistically significant multivariate effect [Wilks’s Λ = 0.560, *F* = 19.194, *p* < 0.001, η^2^_p_ = 0.440 (large effect size)] and a statistically significant between-groups difference [*F* = 12.894, *p* < 0.001, η^2^_p_ = 0.044 (small effect size)].

**Figure 2:**
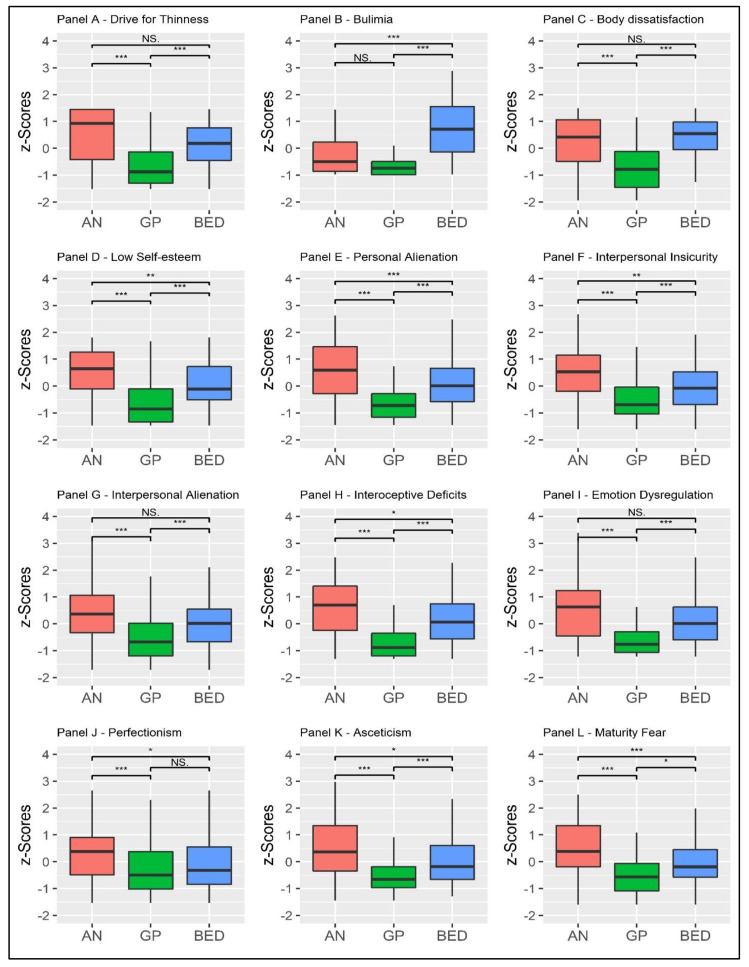
Between-group means comparison. Values are expressed as z-scores. *Note*: * *p*_bonf_ < 0.50; ** *p*_bonf_ < 0.010; *** *p*_bonf_ < 0.001. AN = participants with anorexia nervosa (*n* = 142); BED = participants with binge eating disorder (*n* = 139); GP = participants from the general population (*n* = 140).

Considering the DT, MANOVA revealed statistically significant differences between the three groups: *F* = 59.045, *p* < 0.001, η^2^_p_ = 0.220 (large effect size)—Figure 2, Panel A. Considering the B scale, MANOVA showed statistically significant differences between the three conditions: *F* = 87.590, *p* < 0.001, η^2^_p_ = 0.295 (large effect size)—Figure 2, Panel B. Considering the BD scale, MANOVA revealed statistically significant differences between the three groups: *F* = 75.269, *p* < 0.001, η^2^_p_ = 0.265 (large effect size)—Figure 2, Panel C. Considering the LSE scale, MANOVA showed statistically significant differences between the three conditions: *F* = 66.434, *p* < 0.001, η^2^_p_ = 0.241 (large effect size)—Figure 2, Panel D. Considering the PA scale, MANOVA revealed statistically significant differences between the three groups: *F* = 87.374, *p* < 0.001, η^2^_p_ = 0.295 (large effect size)—Figure 2, Panel E. Considering the II scale, MANOVA showed statistically significant differences between the three conditions: *F* = 44.740, *p* < 0.001, η^2^_p_ = 0.176 (large effect size)—Figure 2, Panel F. Considering the IA scale, MANOVA revealed statistically significant differences between the three groups: *F* = 34.337, *p* < 0.001, η^2^_p_ = 0.141 (large effect size)—Figure 2, Panel G. Considering the ID scale, MANOVA showed statistically significant differences between the three conditions: *F* = 70.248, *p* < 0.001, η^2^_p_ = 0.252 (large effect size)—Figure 2, Panel H. Considering the EmoD scale, MANOVA revealed statistically significant differences between the three groups: *F* = 45.551, *p* < 0.001, η^2^_p_ = 0.179 (large effect size)—Figure 2, Panel I. Considering the P scale, MANOVA showed statistically significant differences between the three conditions: *F* = 14.842, *p* < 0.001, η^2^_p_ = 0.066 (moderate effect size)—Figure 2, Panel J. Considering the A scale, MANOVA revealed statistically significant differences between the three groups: *F* = 45.294, *p* < 0.001, η^2^_p_ = 0.178 (large effect size)—Figure 2, Panel K. Considering the MF scale, MANOVA showed statistically significant differences between the three conditions: *F* = 43.909, *p* < 0.001, η^2^_p_ = 0.174 (large effect size)—Figure 2, Panel L. Bivariate comparisons are reported in Table 2.

### 3.5. Profile Analysis: Flatness—Within-Group Differences

A statistically significant within-groups effect was found: *F* = 17.691, *p* < 0.001, η^2^_p_ = 0.078 (moderate effect size). This result confirmed that there were overall differences in the average values of the dependent variables.

Considering patients with AN, a statistically significant multivariate effect [Wilks’s Λ = 0.585, *F* = 8.460, *p* < 0.001, η^2^_p_ = 0.415 (large effect size)] and a statistically significant within-group differences [*F* = 11.947, *p* < 0.001, η^2^_p_ = 0.075 (moderate effect size)] were found. Considering participants enrolled from the GP, a statistically significant multivariate effect [Wilks’s Λ = 0.670, *F* = 5.787, *p* < 0.001, η^2^_p_ = 0.330 (large effect size)] and a statistically significant within-group differences [*F* = 6.438, *p* < 0.001, η^2^_p_ = 0.044 (small effect size)] were found. Considering patients with AN, a statistically significant multivariate effect [Wilks’s Λ = 0.483, *F* = 12.463, *p* < 0.001, η^2^_p_ = 0.517 (large effect size)] and a statistically significant within-group differences [*F* = 16.072, *p* < 0.001, η^2^_p_ = 0.104 (moderate effect size)] were found.

Detailed results—bivariate comparisons—are reported in Table 3.

## 4. Discussion

The present study employs profile analysis to investigate similitudes and differences in core ED symptoms and general psychological constructs as assessed by the EDI-3 among samples of patients with AN and BED and respondents from the GP.

Results support previous research in suggesting that distinct patterns of symptom presentation exist between the three samples across all the EDI-3 dimensions, but for the first time, clinical samples of patients with AN and BED are directly compared here.

Not surprisingly, both the AN and BED groups separately showed significantly higher scores across the majority of the EDI-3 dimensions than the community sample. Moreover, statically significant differences in specific symptom presentations were observed between the two clinical samples (AN vs. BED).

Of note, higher scores across all the psychological trait scales were detected in the AN group compared to their counterpart, together with significantly higher scores in the drive for thinness ED-specific dimension. Patients with BED showed, instead, a greater tendency to engage in uncontrollable overeating (bulimia) and higher (but not statistically different) scores in the body dissatisfaction dimension compared to the AN group.

Indeed, dissatisfaction with body shape and weight is a key indicator and risk factor for the development of AN and BN [56]; the Diagnostic and Statistical Manual of Mental Disorders, 5th ed. (DSM-5) [1] does not contain a body image-related criterion for BED [3]. Results from this study support those from previous research in which patients with BED showed remarkable concerns about weight and body shape [57], extreme body dissatisfaction, [58] as well as body checking and avoidance behaviors [59]. Additionally, studies exploring the presence of altered body perception in patients with BED concluded that they may either over- [60,61] or underestimate [62,63] their body size. Despite leading to inconsistent conclusions, these findings further indicate a link between distorted body image and its related constructs (i.e., body dissatisfaction) and BED symptomatology. Therefore, the application of body dissatisfaction as a diagnostic indicator seems reasonable but—given the results of this study—only considering other variables associated with higher levels of ED pathology including lower self-esteem.

Indeed, it is unquestionable that low self-esteem is linked with EDs, and a decrease in self-esteem is correlated with poorer body image [64,65], perfectionism [66,67,68], and bulimic symptoms [69,70,71]. However, how these variables are related is not entirely well-defined: is it low self-esteem that makes an individual more susceptible to an ED, or is it the presence of disordered eating patterns that impacts the individual’s self-esteem?

In a review of the literature, Ghaderi (2010) concluded that low self-esteem, along with other factors, not only puts an individual at greater risk for the development of an ED but also serves as a maintaining factor [72]. Indeed, individuals with higher levels of disordered eating behaviors displayed higher levels of overall dissatisfaction with themselves, their appearance, and their family relationships in many studies [73,74].

Self-esteem is «a sense of contentment and self-acceptance that results from a person’s appraisal of their worth, attractiveness, competence, and ability to satisfy their aspirations» [72].

Given this definition, it is clear to see that self-esteem is multifaceted. Similarly, the development and maintenance of EDs are complex and involve factors such as family and cultural environments, history of dieting and/or food addiction, developmental stage, and relational, emotional, and spiritual factors [74,75,76].

The research found that the development of AN and BN is predicted by perfectionistic tendencies and body dissatisfaction only among women with low self-esteem, whereas women showing higher self-esteem did not develop anorexic and/or bulimic symptoms [66,77,78].

The results from this study support the occurrence of low self-esteem in both clinical samples and partially support the cognitive-behavioral theorization of eating behaviors as driven and maintained by attempts to adhere to extreme dietary rules; the results also seem to reflect similar—albeit different—tendencies in AN and BED.

The ideal thinness—a noticeable trend in society—would drive those individuals that also scored significantly higher in the EDI-3 perfectionism dimension to cope by striving to become the thinnest they can—thus anticipating the onset of AN. Furthermore, if these factors are buffered by low self-esteem, an intense drive for thinness would follow—functioning as maintaining factors.

Instead, individuals showing less perfectionistic attitudes but still engaging in restrained eating would develop BED symptoms if unable to adhere to extreme dietary rules, and concerns about their ability to control their eating, shape, and weight matched with perceived poor self-control and personal weakness, further encouraging dietary restraint.

Despite coming to interesting conclusions, the present study has a few limitations. First, a single psychometric measure has been used, and its self-report nature may also have affected the reliability of the tool due to the socially desirable response tendency. Moreover, since the EDI—and particularly its body dissatisfaction subscale—was originally designed for underweight to normal-weight populations of individuals with AN and BN, it might fail to detect differences in samples of overweight or obese participants with BED. Second, although this study was based on a solid literature background, the research design was cross-sectional, and, therefore, it is not possible to assess the incidence or make a causal inference. Thus, future research should fill this gap by conducting longitudinal studies or employing stronger methodologies (i.e., randomized control trials) aimed at exploring these constructs over time. Third, while subjects meeting the BED criteria in the present study are distinguishable from patients with AN based on their bulimia subscale scores, the absence of a sample of participants with BN—or patients with obesity without BED—prevents drawing further considerations and research hypotheses for future studies on the etiopathogenesis and maintenance of overeating tendencies. Fourth, the data presented are collected from patients attending a specialist ED rehabilitation program, but there is a plethora of evidence that the majority of those suffering from EDs are not receiving active treatment.

The results from this study are useful for helping clinicians assess target variables while working with patients with EDs, therefore furthering knowledge and enhancing treatment strategies for AN and BED. Specifically, an intervention targeting self-esteem seems particularly helpful to prevent the worsening of the clinical presentation of patients with dysfunctional eating habits. Furthermore, baseline assessment of the presence of body image disturbance in both individuals with AN and higher weight might help support the development of effective treatment to reduce ED symptoms. However, in light of the above limitations, findings from this study must be taken with caution and inspire further research in the field. Future studies might indeed use different, additional self-report measures to corroborate the above-mentioned conclusion. Models of predictive/protective factors for the onset of AN and BED might be also tested in longitudinal research.

## Figures and Tables

**Table 1:** Descriptive statistics of questionnaires (row scores) and correlations between variables.

		Descriptive Statistics				Correlations											Collinearity	
		M	SD	Sk	K	1	2	3	4	5	6	7	8	9	10	11	T	VIF
1	DT	14.25	9.44	0.20	−0.69	-											0.395	2.531
2	B	8.09	8.29	0.92	−0.24	0.372	-										0.684	1.463
3	BD	22.60	11.62	0.30	2.51	0.692	0.497	-									0.416	2.402
4	LSE	10.73	7.33	0.22	−1.08	0.664	0.398	0.644	-								0.293	3.409
5	PA	9.92	6.88	0.60	−0.39	0.581	0.374	0.556	0.784	-							0.249	4.023
6	II	10.49	6.55	0.37	−0.65	0.482	0.250	0.446	0.622	0.645	-						0.426	2.350
7	IA	9.86	5.75	0.46	−0.16	0.452	0.313	0.449	0.639	0.691	0.697	-					0.379	2.637
8	ID	12.36	9.52	0.55	−0.72	0.623	0.403	0.546	0.711	0.776	0.663	0.644	-				0.266	3.757
9	EmoD	7.90	6.51	0.70	−0.36	0.531	0.371	0.426	0.601	0.649	0.504	0.619	0.718	-			0.400	2.498
10	P	8.82	5.73	0.59	−0.36	0.377	0.194	0.248	0.387	0.421	0.347	0.441	0.429	0.436	-		0.679	1.473
11	A	9.16	6.36	0.86	0.14	0.649	0.382	0.556	0.642	0.674	0.548	0.579	0.706	0.651	0.495	-	0.371	2.692
12	MF	12.46	7.80	0.58	−0.28	0.426	0.188	0.378	0.495	0.552	0.403	0.441	0.509	0.471	0.295	0.452	0.641	1.559

Note: each correlation is statistically significant with *p* < 0.001. M = mean; SD = standard deviation; Sk = skewness; T = tolerance; VIF = Variance Inflation Factor; K = Kurtosis. DT = Drive for Thinness; B = Bulimia; BD = Body dissatisfaction; LSE = Low Self-Esteem; PA = Personal Alienation; II = Interpersonal Insecurity; IA = Interpersonal Alienation; ID = Interoceptive Deficits; EmoD = Emotion Dysregulation; P = Perfectionism; A = Ascetism; MF = Maturity fear.

**Table 2:** Between-groups comparison.

		Descriptive Statistics						Group Comparisons					
		AN		GP		BED		AN vs. GP		GP vs. BED		AN vs. BED	
		M	SD	M	SD	M	SD	*t*	*d*	*t*	*d*	*t*	*d*
1	DT	18.58	9.813	8.28	7.177	15.66	7.299	10.321 *	1.198	−7.355 *	−1.020	2.922	0.337
2	B	6.75	7.131	3.40	4.956	14.19	8.398	3.818	0.544	−12.249 *	−1.567	−8.481 *	−0.957
3	BD	25.42	10.294	14.48	10.068	27.49	8.054	8.899 *	1.075	−10.523 *	−1.426	−1.677	−0.223
4	LSE	14.85	6.801	6.10	5.737	11.19	6.609	11.286 *	1.390	−6.525 *	−0.822	4.718 *	0.546
5	PA	14.15	6.964	5.08	3.732	10.49	6.171	12.456 *	1.620	−7.392 *	−1.062	5.016 *	0.556
6	II	13.89	6.598	7.19	5.218	10.35	5.971	9.682 *	1.127	−4.536 *	−0.564	5.113 *	0.564
7	IA	12.27	5.805	7.04	4.673	10.24	5.478	8.600 *	0.992	−5.235 *	−0.629	3.331	0.360
8	ID	17.53	9.853	6.03	5.783	13.45	8.572	11.421 *	1.421	−7.335 *	−1.016	4.040 *	0.441
9	EmoD	10.92	6.795	4.27	4.509	8.47	6.188	9.655 *	1.152	−6.070 *	−0.777	3.547	0.376
10	P	10.82	5.608	7.36	5.319	8.25	5.719	5.718 *	0.634	−1.468	−0.162	4.235 *	0.454
11	A	12.27	7.286	5.74	3.815	9.43	5.672	9.713 *	1.121	−5.456 *	−0.764	4.220 *	0.435
12	MF	16.61	8.054	8.71	6.240	11.99	6.909	9.565 *	1.095	−3.958 *	−0.499	5.576 *	0.614

Note: * = *p*-value is statistically significant with Bonferroni correction; M = mean (raw score); SD = standard deviation (raw score); *t* = independent-sample *t*-test (computed on *z*-scores); *d* = Cohen’s *d* (effect size) for independent groups comparisons (computed on *z*-scores). AN = anorexia nervosa; GP = general population; BED = binge eating disorder; DT = Drive for Thinness; B = Bulimia; BD = Body dissatisfaction; LSE = Low Self-Esteem; PA = Personal Alienation; II = Interpersonal Insecurity; IA = Interpersonal Alienation; ID = Interoceptive Deficits; EmoD = Emotion Dysregulation; P = Perfectionism; A = Ascetism; MF = Maturity fear.

**Table 3:** Within-group comparison.

		AN		GP		BED	
		*t*	*d*	*t*	*d*	*t*	*d*
DT	B	7.137 *	0.599	−0.966	−0.082	−7.219 *	−0.612
	BD	2.480	0.208	0.947	0.080	−3.338	−0.283
	LSE	−1.187	−0.100	−0.018	−0.002	1.070	0.091
	PA	−1.780	−0.149	1.027	0.087	0.824	0.070
	II	−0.697	−0.059	−1.859	−0.157	2.108	0.179
	IA	0.456	0.038	−2.061	−0.174	1.021	0.087
	ID	−0.969	−0.081	0.463	0.039	0.420	0.036
	EmoD	−0.058	−0.005	−1.092	−0.092	0.753	0.064
	P	1.257	0.106	−5.467 *	−0.462	3.056	0.259
	A	−0.352	−0.030	−1.374	−0.116	1.316	0.112
	MF	−0.837	−0.070	−2.212	−0.187	2.563	0.217
B	BD	−4.657 *	−0.391	1.913	0.162	3.881 *	0.329
	LSE	−8.325 *	−0.699	0.948	0.080	8.289 *	0.703
	PA	−8.917 *	−0.748	1.994	0.168	8.043 *	0.682
	II	−7.835 *	−0.657	−0.892	−0.075	9.327 *	0.791
	IA	−6.682 *	−0.561	−1.095	−0.093	8.240 *	0.699
	ID	−8.106 *	−0.680	1.429	0.121	7.639 *	0.648
	EmoD	−7.195 *	−0.604	−0.126	−0.011	7.972 *	0.676
	P	−5.880 *	−0.493	−4.500 *	−0.380	10.275 *	0.872
	A	−7.489 *	−0.628	−0.408	−0.034	8.535 *	0.724
	MF	−7.974 *	−0.669	−1.246	−0.105	9.782 *	0.830
BD	LSE	−3.667 *	−0.308	−0.965	−0.082	4.408 *	0.374
	PA	−4.260 *	−0.357	0.080	0.007	4.162 *	0.353
	II	−3.177	−0.267	−2.806	−0.237	5.446 *	0.462
	IA	−2.024	−0.170	−3.009	−0.254	4.359 *	0.370
	ID	−3.449 *	−0.289	−0.484	−0.041	3.758 *	0.319
	EmoD	−2.537	−0.213	−2.039	−0.172	4.091 *	0.347
	P	−1.222	−0.103	−6.414 *	−0.542	6.394 *	0.542
	A	−2.832	−0.238	−2.321	−0.196	4.654 *	0.395
	MF	−3.317	−0.278	−3.160	−0.267	5.901 *	0.501
LSE	PA	−0.593	−0.050	1.045	0.088	−0.246	−0.021
	II	0.490	0.041	−1.841	−0.156	1.038	0.088
	IA	1.643	0.138	−2.044	−0.173	−0.048	−0.004
	ID	0.218	0.018	0.481	0.041	−0.650	−0.055
	EmoD	1.130	0.095	−1.074	−0.091	−0.316	−0.027
	P	2.445	0.205	−5.449 *	−0.460	1.986	0.168
	A	0.836	0.070	−1.356	−0.115	0.246	0.021
	MF	0.351	0.029	−2.195	−0.185	1.493	0.127
PA	II	1.083	0.091	−2.886	−0.244	1.284	0.109
	IA	2.236	0.188	−3.089	−0.261	0.197	0.017
	ID	0.811	0.068	−0.564	−0.048	−0.404	−0.034
	EmoD	1.723	0.145	−2.119	−0.179	−0.071	−0.006
	P	3.038	0.255	−6.494 *	−0.549	2.232	0.189
	A	1.429	0.120	−2.401	−0.203	0.492	0.042
	MF	0.943	0.079	−3.240	−0.274	1.739	0.148
II	IA	1.153	0.097	−0.203	−0.017	−1.087	−0.092
	ID	−0.272	−0.023	2.322	0.196	−1.688	−0.143
	EmoD	0.640	0.054	0.767	0.065	−1.355	−0.115
	P	1.955	0.164	−3.608 *	−0.305	0.948	0.080
	A	0.346	0.029	0.485	0.041	−0.792	−0.067
	MF	−0.139	−0.012	−0.354	−0.030	0.455	0.039
IA	ID	−1.425	−0.120	2.525	0.213	−0.601	−0.051
	EmoD	−0.513	−0.043	0.969	0.082	−0.268	−0.023
	P	0.802	0.067	−3.405	−0.288	2.035	0.173
	A	−0.807	−0.068	0.688	0.058	0.294	0.025
	MF	−1.292	−0.108	−0.151	−0.013	1.542	0.131
ID	EmoD	0.912	0.076	−1.555	−0.131	0.333	0.028
	P	2.227	0.187	−5.930 *	−0.501	2.636	0.224
	A	0.618	0.052	−1.837	−0.155	0.896	0.076
	MF	0.132	0.011	−2.676	−0.226	2.143	0.182
EmoD	P	1.315	0.110	−4.374 *	−0.370	2.303	0.195
	A	−0.294	−0.025	−0.282	−0.024	0.562	0.048
	MF	−0.779	−0.065	−1.120	−0.095	1.810	0.154
P	A	−1.609	−0.135	4.093	0.346	−1.740	−0.148
	MF	−2.094	−0.176	3.254	0.275	−0.493	−0.042
A	MF	−0.485	−0.041	−0.838	−0.071	1.247	0.106

Note: * = *p*-value is statistically significant with Bonferroni correction; M = mean (raw score); SD = standard deviation (raw score); *t* = paired-sample *t*-test (computed on *z*-scores); *d* = Cohen’s *d* (effect size) for dependent groups comparisons (computed on *z*-scores). AN = anorexia nervosa; GP = general population; BED = binge eating disorder; DT = Drive for Thinness; B = Bulimia; BD = Body dissatisfaction; LSE = Low Self-Esteem; PA = Personal Alienation; II = Interpersonal Insecurity; IA = Interpersonal Alienation; ID = Interoceptive Deficits; EmoD = Emotion Dysregulation; P = Perfectionism; A = Ascetism; MF = Maturity Fear.

## Data Availability

Due to privacy restrictions, the dataset used in the current study is available from the corresponding author upon reasonable request.
